# Comparative genomics and metabolomics analysis of *Riemerella anatipestifer* strain CH-1 and CH-2

**DOI:** 10.1038/s41598-020-79733-w

**Published:** 2021-01-12

**Authors:** Jibin Liu, Anchun Cheng, Mingshu Wang, Mafeng Liu, Dekang Zhu, Qiao Yang, Ying Wu, Renyong Jia, Shun Chen, Xinxin Zhao, Shaqiu Zhang, Juan Huang, Xumin Ou, Sai Mao, Qun Gao, Xingjian Wen, Ling Zhang, Yunya Liu, Yanling Yu, Bin Tian, Leichang Pan, Mujeeb Ur Rehman, Xiaoyue Chen

**Affiliations:** 1 Institute of Preventive Veterinary Medicine, College of Veterinary Medicine, Sichuan Agricultural University, Chengdu, 611130 Sichuan China; 2 Research Center of Avian Diseases, College of Veterinary Medicine, Sichuan Agricultural University, Chengdu, 611130 Sichuan China; 3 Key Laboratory of Animal Disease and Human Health of Sichuan Province, Sichuan Agricultural University, Wenjiang, Chengdu, 611130 Sichuan China

**Keywords:** Metabolomics, Bacteria, Pathogens

## Abstract

*Riemerella anatipestifer* is a major pathogenic microorganism in poultry causing serositis with significant mortality. Serotype 1 and 2 were most pathogenic, prevalent, and liable over the world. In this study, the intracellular metabolites in *R. anatipestifer* strains RA-CH-1 (serotype 1) and RA-CH-2 (serotype 2) were identified by gas chromatography-mass spectrometer (GC–MS). The metabolic profiles were performed using hierarchical clustering and partial least squares discriminant analysis (PLS-DA). The results of hierarchical cluster analysis showed that the amounts of the detected metabolites were more abundant in RA-CH-2. RA-CH-1 and RA-CH-2 were separated by the PLS-DA model. 24 potential biomarkers participated in nine metabolisms were contributed predominantly to the separation. Based on the complete genome sequence database and metabolite data, the first large-scale metabolic models of *i*JL463 (RA-CH-1) and *i*DZ470 (RA-CH-2) were reconstructed. In addition, we explained the change of purine metabolism combined with the transcriptome and metabolomics data. The study showed that it is possible to detect and differentiate between these two organisms based on their intracellular metabolites using GC–MS. The present research fills a gap in the metabolomics characteristics of *R. anatipestifer*.

## Introduction

*Riemerella anatipestifer* (RA) is a Gram-negative pathogen with a capsule and belongs to the family of *Flavobacteriaceae*. Previous studies have reported that the genetic modification in RA was occurred simply and rapidly by the natural transformation^1–8^. RA infection is a major epidemic disease of fowls, which causes septicemia and is characterized by serious fibrinous polyserositis. So far, at least 21 serotypes RA were reported in the world^9–23^. Serotypes 1 and 2 of RA were the dominating serotypes in China. The virulence and metabolic capability in two serotypes have seen some differences across and within serotypes. While some research has been carried out on many pathogenicity and resistance factors in the two strains^24–28^ data about the metabolic pathways required for growth and infection, particularly the natural utilization of carbon and nitrogen sources has remained unclear.

Metabolism plays an important role in host–microbe interactions whether acute or persistent infections^29^. Researches on the types and contents of metabolites, as the final products of gene expression in the organism, are a critical supplement to genomics and proteomics research. Previous studies have shown that RA cannot grow on common nutrition agar or MacConkey nutrition agar and is slightly more challenging to grow in vitro. Unlike many other *Flavobacteriaceae* family germs, RA cannot ferment carbohydrates, although some strains can produce acid by glucose, maltose, inositol and fructose^30^. Compared with other gram-negative bacterial infection of the domestic birds, the reaction of oxidase and catalase in RA were positively tested. Moreover, acid and alkaline phosphatase, gelatinase, esterase C4, ester lipase C8, α-glucosidase and leucine-, valine- and cysteine-arylamidases phosphoamidase are also found in RA^31–33^. Although metabolites are not the gene direct products, the concentrations of metabolites are a strong correlation with enzymatic activity.

As biological scientific technology advances, high-throughput data has reshaped many approaches to studying the biology of an organism. For model organisms such as *E. coli*, the combination of genomics, proteomics and metabolomics are a critical supplement to biology and physiology^34^. As the whole genomic sequences and transcriptomic data of RA continue to grow^35–39^, the results make it possible to reconstruct the metabolic network and assess the metabolic capabilities and fitness of RA. RA wild type strain CH-1 (RA-CH-1) and CH-2 (RA-CH-2) were the representatives of the serotype 1 and 2, respectively^40^. These two strains are multi-drug resistant strains, which had high resistance of tetracyclines, macrolides, sulfonamides, aminoglycoside antibiotics, chloramphenicol and quinolones^41^. Metabolomics, as a rapidly evolving tool, is used to identify and quantify small molecule metabolites that define the metabolic status of an organism^42^. Untargeted, semi-targeted and targeted metabolomics were the three analytical approaches applied in metabolomics. Unlike targeted metabolomics, untargeted metabolomics is to qualitatively determine the chemical profiles of biological samples^43^. Gas chromatography–mass spectrometry (GC–MS) is one of the most commonly used techniques for untargeted metabolomics due to its high sensitivity and selectivity in untargeted metabolic profiling^44^. In this study, we introduce a method based on untargeted metabolomics for metabolic profiling in RA. Application of this method to two species structured a database of metabolites depending on a group of models from different metabolic pathways. Our study applied the metabolomics platform to elucidate the metabolic characteristics of RA. Subsequently, we used the metabolomics and genomic data to build a genome-scale metabolic network. This study is the first to report a reconstructed genome-scale metabolic model for RA based on the available genomics and metabolomics data. We believe these data sets of RA further illustrate the use of metabolic profiling as an additional tool and offer some important insights into metabolism*.*

## Results

### The metabolite profile of two RA species

**Figure 1:**
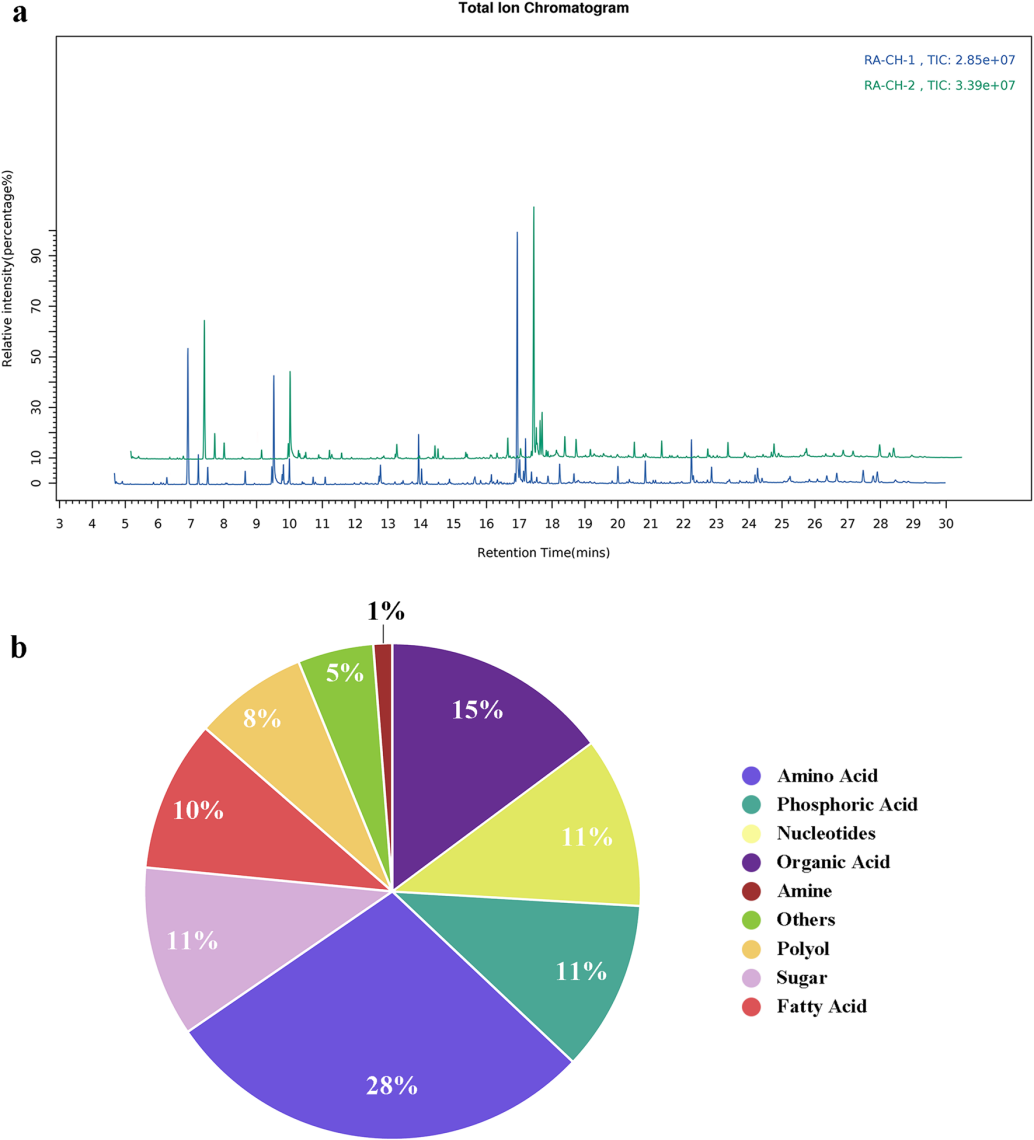
The metabolite profile of two RA species by GC–MS. (**a**) GC–MS total ion-chromatograms (TIC) of the typical metabolome samples from RA-CH-1 (blue) and RA-CH-2 (green) cultivated in TSB medium and sampled in the stationary growth phase. (**b**) Classification of 81 identified metabolites identified in RA.

To find more intracellular metabolites in RA, a non-target metabolomics approach was used for RA metabolic profiling. Figure 1a showed that the total ion chromatogram (TIC) of samples from the two RA strains, thus illustrating the significant variations of metabolic profiling. 81 different metabolites were confirmed as listed in Table 1. 39 compounds were identified by pure standard compounds and MS-library match. These compounds were divided into nine classes of chemical substances, including nucleotides, organic acids, amino acids, phosphates, sugars, fatty acids, amines, polyols and others (nicotinamide, urea, parabanic acid and 2,4,6-tritert-butylbenzenethiol). The chemical classes of amino acids, organic acids, nucleotides, phosphates, sugars, fatty acids accounted for 28%, 15%, 11%, 11%, 11% and 10% of all identified metabolites, respectively. Relatively few polyols (8%) and putrescine involved in amine (1%) were identified (Fig. 1b).

**Table 1:** Classification of identified metabolites.

Metabolite	TMS-form	Classification	RI	m/z	rt/min
Putrescine	Putrescine (3 TMS)	Amine	1459.2	174.1	12.0
Alanine*	Alanine (2 TMS)	Amino acid	1100.2	116.1	6.9
Asparagine*	Asparagine (3 TMS)	Amino acid	1674.4	116	14.6
Aspartic acid*	Aspartic acid (3 TMS)	Amino acid	1522.7	232.1	12.8
Cysteine*	Cysteine (3 TMS)	Amino acid	1558.5	220.1	13.2
Glutamic acid*	Glutamic acid (3 TMS)	Amino acid	1620.8	246.1	14.0
Glutamine	Glutamine (3 TMS)	Amino acid	1776	156.1	15.7
Glycine*	Glycine (3 TMS)	Amino acid	1306	174.1	10.0
Homoserine	Homoserine (3 TMS)	Amino acid	1451.6	218.1	11.9
Isoleucine*	Isoleucine (2 TMS)	Amino acid	1293	158.1	9.8
Leucine*	Leucine (2 TMS)	Amino acid	1271.3	158.1	9.5
Lysine*	Lysine (3 TMS)	Amino acid	1852.8	174.1	16.5
Methionine*	Methionine (2 TMS)	Amino acid	1519.1	176.1	12.8
N-Acetylglutamic acid	N-Acetylglutamic acid (2 TMS)	Amino acid	1527.7	84.02	12.8
Ornithine*	Ornithine (4 TMS)	Amino acid	1823.5	142.1	16.2
Phenylalanine*	Phenylalanine (2 TMS)	Amino acid	1629	192.1	14.0
Proline*	Proline (2 TMS)	Amino acid	1295.7	142.1	9.8
Pyroglutamic acid*	Pyroglutamic acid (2 TMS)	Amino acid	1522.9	156.1	12.8
Serine*	Serine (3 TMS)	Amino acid	1362.5	204.1	10.7
Suberyl glycine	Suberyl glycine (3 TMS)	Amino acid	1637.1	188	14.1
Threonine*	Threonine (3 TMS)	Amino acid	1389.7	117	11.1
Tryptophan*	Tryptophan (3 TMS)	Amino acid	2214	202.1	19.9
Tyrosine*	Tyrosine (3 TMS)	Amino acid	1944.6	218.1	17.4
Valine*	Valine (2 TMS)	Amino acid	1214.8	144.1	8.7
Dodecanoic acid	Dodecanoic acid (1 TMS)	Fatty acid	1642.8	117	14.2
Heptadecanoic acid	Heptadecanoic acid (1 TMS)	Fatty acid	2095.2	327.3	18.8
Heptanoic acid	Heptanoic acid (1 TMS)	Fatty acid	1169.4	144.1	8.0
Hexadecanoic acid*	Hexadecanoic acid (1 TMS)	Fatty acid	2033.4	117	18.3
Nonanoic acid	Nonanoic acid (1 TMS)	Fatty acid	1352.9	117	10.6
Octadecanoic acid	Octadecanoic acid (1 TMS)	Fatty acid	2229.9	117	20.0
Pentadecanoic acid	Pentadecanoic acid (1 TMS)	Fatty acid	1900.6	299.3	17.0
Tetradecanoic acid	Tetradecanoic acid (1 TMS)	Fatty acid	1801.4	117	15.9
Adenine	Adenine (2 TMS)	Nucleotides	1870.2	264.1	16.7
Adenosine	Adenosine (4 TMS)	Nucleotides	2652	230.1	23.4
Cytosine	Cytosine (2 TMS)	Nucleotides	1525.1	254.1	12.8
Guanine	Guanine (4 TMS)	Nucleotides	2133.1	352.2	19.2
Guanosine	Guanosine (5 TMS)	Nucleotides	2796.9	324.1	24.4
Hypoxanthine	Hypoxanthine (2 TMS)	Nucleotides	1807.6	265.1	16.0
Thymine	Thymine (2 TMS)	Nucleotides	1401.5	255.1	11.3
Uracil	Uracil (2 TMS)	Nucleotides	1338.1	241.1	10.4
Uridine	Uridine (3 TMS)	Nucleotides	2462.2	217.1	21.9
α-Ketoglutaric acid*	alpha-ketoglutaric acid (1MEOX) (2TMS)	Organic acid	1576.5	198	13.4
Citramalic acid	Citramalic acid (3 TMS)	Organic acid	1559.6	247.1	13.2
Citric acid	Citric acid (4 TMS)	Organic acid	1830.1	273.1	16.2
Fumaric acid	Fumaric acid (2 TMS)	Organic acid	1341.4	245.1	10.5
Glyceric acid	Glyceric acid (3 TMS)	Organic acid	1332.9	189	10.3
Glycolic acid	Glycolic acid (2 TMS)	Organic acid	1071.9	177	6.5
Lactic acid	Lactic acid (2 TMS)	Organic acid	1057.8	117	6.3
Maleic acid	Maleic acid (2 TMS)	Organic acid	1301.6	245	9.9
Malic acid*	Malic acid (3 TMS)	Organic acid	1490.5	233.1	12.4
Pipecolic acid	Pipecolic acid (N,O-TMS)	Organic acid	1603.5	156.1	13.8
Pyruvic acid	Pyruvic acid (1 MEOX; 1 TMS)	Organic acid	1046.2	174	6.1
Succinic acid	Succinic acid (2 TMS)	Organic acid	1308.9	247.1	10.0
Adenosine-5-monophosphate	Adenosine-5-monophosphate(5 TMS)	Phosphates	3097.9	169	26.7
Fructose-6-phosphate*	Fructose-6-phosphate (1MEOX) (6 TMS)	Phosphates	2343.3	315.1	21.0
Glucose-6-phosphate*	Glucose-6-phosphate (1MEOX) (6 TMS)	Phosphates	2357.6	387.2	21.1
Glyceric acid-3-phosphate*	Glyceric acid-3-phosphate (4 TMS)	Phosphates	1820.1	227	16.1
Glycerol-2-phosphate	Glycerol-2-phosphate (4 TMS)	Phosphates	1738.4	243	15.3
Glycerol-3-phosphate*	Glycerol-3-phosphate (4 TMS)	Phosphates	1773.5	299.1	15.6
Monomethylphosphate	Monomethylphosphate (2 TMS)	Phosphates	1177.9	241	8.1
Myo-inositol-1-phosphate	Myo-Inositol-1-phosphate (7 TMS)	Phosphates	2421.2	299.1	21.6
Phosphoric acid*	phosphoric acid (3 TMS)	Phosphates	1275.4	299.1	9.5
1-Monohexadecanoylglycerol	1-Monohexadecanoylglycerol (2 TMS)	Polyol	2583.3	371.3	22.9
1-Monooctadecanoylglycerol	1-Monooctadecanoylglycerol (2 TMS)	Polyol	2775.8	399.3	24.3
2-Monopalmitoylglycerol	2-Monopalmitoylglycerol (2 TMS)	Polyol	2549.7	129	22.6
2-Monostearoylglycerol	2-Monostearoylglycerol (2 TMS)	Polyol	2740.5	129	24.0
Eicosanol	Eicosanol (1 TMS)	Polyol	2328.6	355.3	20.9
Sorbitol*	Sorbitol (6 TMS)	Polyol	1960.8	319.1	17.6
Arabinose*	Arabinose (1MEOX) (4 TMS)	Sugar	1692.4	103	14.8
Erythrose	Erythrose (1MEOX) (3TMS)	Sugar	1461	117	12.0
Fructose*	Fructose (1MEOX) (5 TMS)	Sugar	1903.2	319.2	17.2
Galactose*	Galactose (1MEOX) (5 TMS)	Sugar	1920.2	103	17.2
Glucopyranose	Glucopyranose (5 TMS)	Sugar	1997.2	204.1	17.9
Glucose*	Glucose (1MEOX) (5 TMS)	Sugar	1939.3	319.2	17.3
Isomaltose*	Isomaltose (1MEOX) (8 TMS)	Sugar	2924.9	361.2	25.3
Sucrose*	Sucrose (8 TMS)	Sugar	2687.1	361.2	23.6
Trehalose*	Trehalose (8 TMS)	Sugar	2794.6	361.2	24.4
2,4,6-Tritert-butylbenzenethiol	2,4,6-Tritert-butylbenzenethiol	Others	1542.6	263.2	13.0
Nicotinamide	Nicotinamide (1 TMS)	Others	1478.8	179.0	12.3
Parabanic acid	Parabanic acid (2 TMS)	Others	1499	100.0	12.5
Urea	Urea (2 TMS)	Others	1233.7	189.1	8.9

*RI* retention index, *m/z* mass to charge ratio, *rt* retention time.

*Metabolites were identified by comparison of pure standard compounds and MS-library.

### Metabolites correlation analysis in RA-CH-1 and RA-CH-2

**Figure 2:**
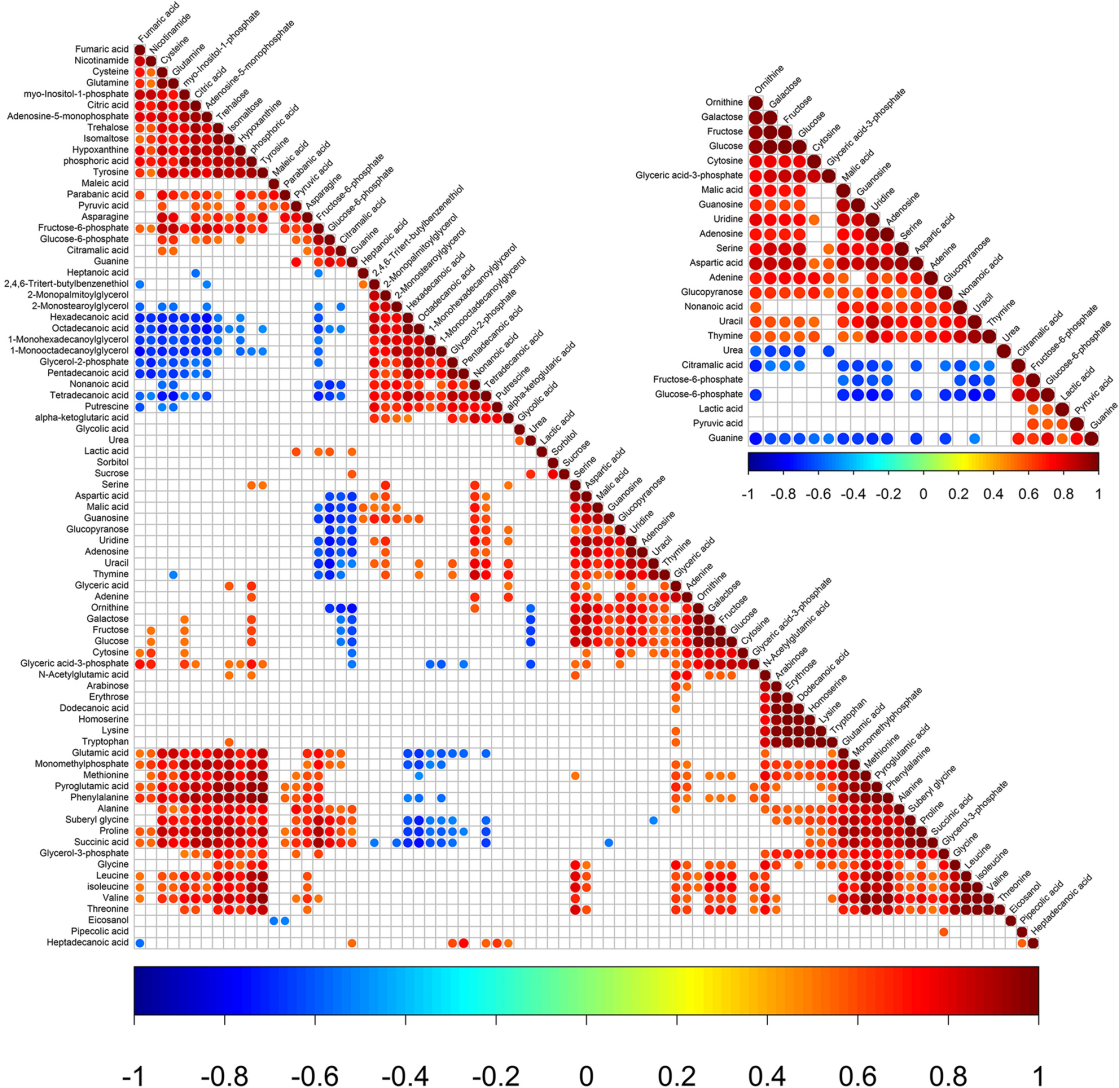
Metabolite-metabolite correlation analysis. Blank squares: p > 0.05. Marked with red or blue (p < 0.05) are the significant metabolite-metabolite correlations. Positive correlations are shown in red; negative correlations are shown in blue.

Correlation analysis was performed to search for the metabolite–metabolite potential relationships in these two organisms. The results allowed the identified chemicals concerning one other. Particularly, the metabolite–metabolite correlations of the differential metabolites were compared in two sample combinations. As shown in Fig. 2, 1053 of 3240 metabolite–metabolite correlations found a statistically correlation (p < 0.05). Out of 1053 statistical correlations, 874 had a significant positive correlation and 179 were negative. The same chemical class of metabolites was found to have positive correlations. Most of the amino acids and the polyols seem to be negatively correlated by comparison to other metabolites. Meanwhile, many fatty acids and amino acids including hexadecanoic acid, cysteine, octadecanoic acid, proline, pentadecanoic acid, glutamic acid, suberyl glycine and tetradecanoic acid found a significant inverse correlation with other metabolites. However, most of the organic acids and amino acids had positive correlations in comparison to other metabolites.

### Construction and application of the metabolic models in RA

**Figure 3:**
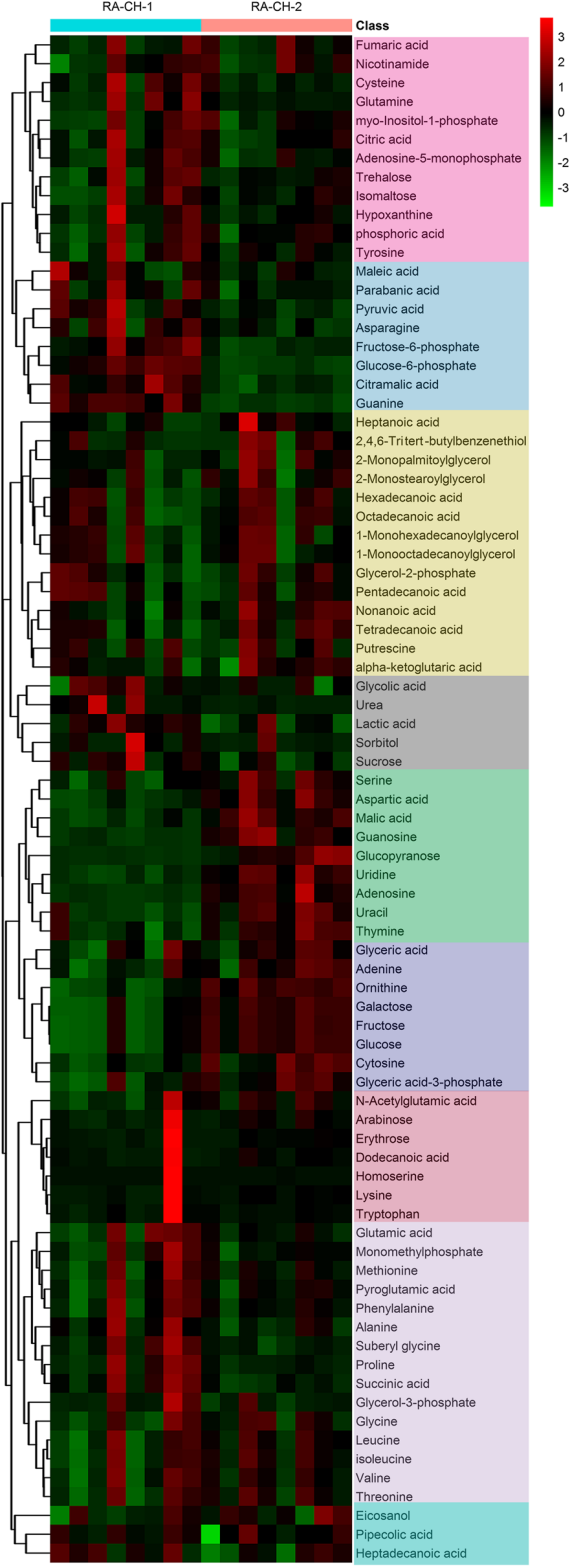
Heatmap of total metabolites in RA-CH-1 and RA-CH-2. Colors represent fold-change values between each line and the samples. Red squares in the heat map indicate increases of intracellular metabolite concentration, while green squares indicate decreases of intracellular metabolite concentration. Fold-change values were log2-transformed. Both columns (samples) and rows (metabolites) were subjected to hierarchical clustering analysis (HCA).

To insight the function and the relationship of the metabolites, the 81 identified metabolites were classified by hierarchical clustering analysis (HCA). HCA results showed that metabolites were clustered in 9 groups (Fig. 3). Metabolites with similar metabolic patterns have similar functions and participate in the same metabolic pathway. We identified 56 metabolites involved in 7 main metabolisms by the Kyoto Encyclopedia of Genes and Genomes (KEGG) database. The 7 main metabolisms include amino acid metabolisms, carbohydrate metabolisms, energy metabolisms, lipid metabolisms, nucleotide metabolisms, cofactors and vitamin metabolisms and biosynthesis of secondary metabolites.

In an attempt to understand the complex interactions between metabolites and gene products in RA, we constructed the draft genome-scale metabolic (GSM) model of the two organisms based on genomic and metabolite data. The formulation of biomass components directly influences the essentiality of each gene. The available relative abundances of RA were DNA, RNA, proteins, cell wall, lipids and cofactor (Supplementary Table S1). The models were gap-filled in simulated media to force a minimum flux of 0.1 through the bio1 reaction. 16 new reactions were added to the draft RA models, while 1 existing reactions were made reversible (Supplementary Table S2). As shown in Table 2, the GSM model of RA-CH-1 (*i*JL463) consists of 788 reactions, 841 metabolites and 463 genes, while the GSM model of RA-CH-2 (*i*DZ470) consists of 801 reactions, 862 metabolites and 470 genes. No mass imbalance was found at neutral pH. A detailed description of the models containing all network reactions was detailed in the supplemental data (*i*JL463 in Supplementary Table S3 and *i*DZ470 in Supplementary Table S4). Most of the reactions are broadly concentrated in lipid, amino acid, and carbohydrate metabolism. Flux balance analysis (FBA) of *i*JL463 and *i*DZ470 reveals a diversity of the predicted metabolites that are essential for growth in RA (see Supplementary Tables S5, S6).

**Table 2:** Features of the genome and genome-scale metabolic model in RA-CH-1 and RA-CH-2.

Features	*i*JL463/RA-CH-1	*i*DZ470/RA-CH-2
Total genome size (bp)	2.31 M	2.17 M
Open reading frames (ORFs)	2207	2050
ORFs included in the model	463	470
Reactions included in the model	788	801
Reactions assigned with ORFs	652	666
Non-enzymatic reactions	136	135
Unique reactions	10	23
Metabolites	841	862

### Multivariate analysis

To identify strain differences, PCA and PLS-DA were used to analyze the preprocessed GC–MS datasets (see Supplementary Fig. S2 online). The PCA model reveals the general metabolic information and visually eliminates abnormal sample data. All samples from the two organisms appeared in the Hotelling T2 95% confidence, suggesting that all the samples can be used for further analysis. A PCA model was described by the parameters (R^2^X = 0.816, Q^2^ = 0.55). The 1st principal component (PC1) retains maximum variation (36.2%) according to the datasets. The 2nd component (PC2) retains 24.1% of the variance. When PC1 and PC2 have been derived, the two strains were separated from each other. The position of each metabolite is correlated to the contributor of PC1 and PC2 which is visible in Supplementary Table S7 online. Although the score plot should not be used to infer group separation, it may reveal structure (e.g. subgroups) within a group.

To further separate the groups, supervised PLS-DA was carried out by fitting tested samples. By PLS-DA analysis, excellent separation of the groups of RA-CH-1and RA-CH-2 was achieved, suggesting significant differences in metabolites between the two groups. Subsequently, a cluster of 100 permutated models from two components was visualized using validation plots. The quality of the supervised models and the goodness of fit were evaluated by the values of R^2^ and Q^2^. The values of R^2^ and Q^2^ in the exact test were below the original ones, which indicated that the model was reliable. Moreover, the value of Q^2^ =  − 0.299 was obtained, indicating that the model was not over-fit.

### Metabolic variations in RA-CH-1 and RA-CH-2

**Figure 4:**
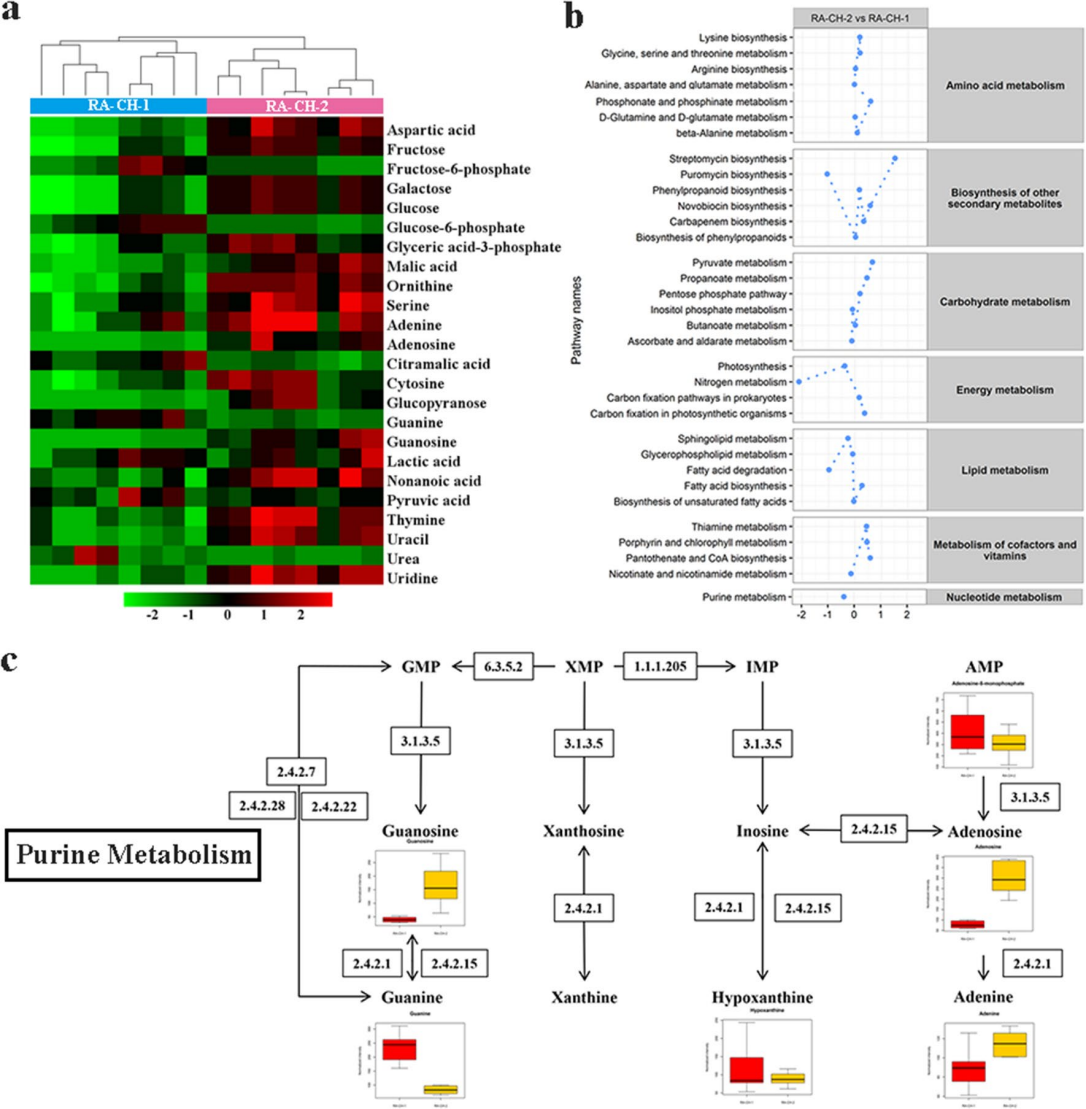
Comparison of network utilization in RA-CH-1 and RA-CH-2. (**a**) HAC of 24 biomarkers in two RA strains. RA-CH-1 (8 samples) and RA-CH-2 (8 samples) belonged to two subgroups. (**b**) Activities of RA metabolic pathways according to comparisons between two RA strains. The activity scores (AS) for each pathway were calculated using the PAPi algorithm. PAPi calculates an AS for each metabolic pathway listed in the KEGG database based on the number of metabolites identified from each pathway and their relative abundances. Related pathways are grouped according to their cellular metabolism and only pathways with statistically significant differences in activity (p < 0.05 by ANOVA) are shown. (**c**) The pathway of Purine metabolism in RA.

To analyze the contents of metabolites detected in the two organisms, 24 potential biomarkers were identified based on the variable importance plot (VIP) > 1.0 values and p values (p < 0.05). The detailed information on their identification is listed in Table 3. As shown in the hierarchical clustering heat map (Fig. 4a), the HCA results showed significant variation between the two organisms. 7 metabolites were detected at higher concentrations in RA-CH-1 samples than RA-CH-2 samples, while 17 metabolites had lower contents in RA-CH-1 samples than RA-CH-2 samples. The 7 higher metabolites included urea, glucose-6-phosphate, citramalic acid, guanine, fructose-6-phosphate, pyruvic acid and lactic acid, while the 17 lower metabolites included 7 nucleotides, 4 sugars, 3 amino acids, 1 organic acid, 1 phosphate and 1 fatty acid. Besides, we found that there was a strong correlation among 24 biomarkers.

**Table 3:** Differential intracellular metabolites between RA-CH-1 and RA-CH-2 groups.

Metabolites	Mean ± SD		VIP	p value^a^	log_2_(FC)^b^
	RA-CH-1	RA-CH-2			
Adenine	88.54 ± 19.43	110.03 ± 22.20	1.06	*	0.31
Adenosine	77.82 ± 17.26	338.12 ± 154.50	1.72	***	2.12
Aspartic acid	525.50 ± 106.35	1021.23 ± 264.38	1.74	***	0.96
Citramalic acid	51.22 ± 19.92	15.09 ± 8.07	1.72	***	− 1.76
Cytosine	22.21 ± 6.94	40.20 ± 13.32	1.47	*	0.86
Fructose	1023.72 ± 907.79	2599.22 ± 467.83	1.66	**	1.34
Fructose-6-phosphate	66.38 ± 29.12	25.60 ± 8.21	1.56	**	− 1.37
Galactose	176.68 ± 144.09	432.91 ± 76.62	1.68	**	1.29
Glucopyranose	350.48 ± 68.97	1858.82 ± 1062.14	1.60	***	2.41
Glucose	194.45 ± 168.22	488.47 ± 84.99	1.67	**	1.33
Glucose-6-phosphate	86.96 ± 22.83	24.40 ± 6.82	1.96	***	− 1.83
Glyceric acid-3-phosphate	53.68 ± 33.34	92.06 ± 25.08	1.25	*	0.78
Guanine	233.34 ± 49.57	83.22 ± 14.72	1.99	***	− 1.49
Guanosine	39.83 ± 9.77	165.73 ± 74.72	1.72	***	2.06
Lactic acid	494.68 ± 92.30	375.54 ± 128.45	1.08	*	− 0.40
Malic acid	26.19 ± 9.95	70.79 ± 22.97	1.76	**	1.43
Nonanoic acid	10.33 ± 2.54	14.19 ± 3.16	1.28	*	0.46
Ornithine	935.73 ± 335.20	1984.35 ± 281.19	1.92	***	1.08
Pyruvic acid	227.56 ± 70.23	152.76 ± 21.49	1.34	*	− 0.57
Serine	939.73 ± 223.20	1287.73 ± 278.92	1.30	*	0.45
Thymine	42.06 ± 11.39	60.38 ± 11.39	1.43	*	0.52
Uracil	285.50 ± 94.70	488.10 ± 109.47	1.59	**	0.77
Urea	616.48 ± 729.53	72.77 ± 56.60	1.07	**	− 3.08
Uridine	135.27 ± 14.28	225.75 ± 43.44	1.82	***	0.74

^a^*p < 0.05; **p < 0.01; ***p < 0.001.

^b^FC: fold change in RA-CH-2/RA-CH-1.

To compare metabolic pathway activities from metabolite profiles between the two organisms, we have correlated metabolomics datasets and metabolic pathways activities using Pathway Activity Profiling (PAPi)^45^. A variety of metabolic pathways participated in the metabolism of amino acids, secondary metabolites, carbon, energy, lipids, cofactors and nucleotides (Fig. 4b). As compared with RA-CH-2, RA-CH-1 did not display much difference in the metabolism of amino acids. However, among secondary metabolites, streptomycin biosynthesis, phenylpropanoid biosynthesis, novobiocin biosynthesis and carbapenem biosynthesis were up-regulated in RA-CH-2. These metabolisms are essential for RA adapted to their environment and improve the survivability of RA against abiotic or biotic stresses. The metabolism of cofactors and vitamins was up-regulated in RA-CH-2 compared to RA-CH-1. This indicated that the metabolic compounds related to this pathway were active in RA-CH-2. Most subgroups of carbohydrate metabolism in RA-CH-2 were up-regulated, suggesting that carbohydrates biosynthesized supply energy for fatty acid metabolism. For example, pyruvate, propanoate and pentose phosphate metabolism were all upregulated in RA-CH-2, indicating that their metabolism was activated.

To observe the metabolite-by-metabolite differences for compounds, nucleotides in two strains were with the maximum changes compared to other metabolites, but purine metabolism did not change a lot (0.39-fold higher in RA-CH-2). Figure 4c represents a schematic of purine metabolism. AMP is converted to adenosine by 5′-nucleotidase (EC 3.1.3.5) and then to adenine by Purine nucleoside phosphorylase (PNP, EC 2.4.2.1). The concentration of Adenosine in RA-CH-2 was two-fold higher than that in RA-CH-1, while the concentration of Adenine in RA-CH-2 was 0.3-fold higher in RA-CH-1.To explore whether differential expression of particular genes would cause reaction flux changes, the reactions associated with genes change were check by the transcriptome analysis (see Supplementary Table S8), the up-regulation of Adenosine in RA-CH-2 could be the reason for the level of 5′-nucleotidase expression significantly increased (1.73-fold change) compared with that in RA-CH-1. Meanwhile, the level of Purine nucleoside phosphorylase expression was significantly reduced (0.86-fold change) in RA-CH-2.

## Discussion

Metabolomics is a group of indicators for high-throughput detection and data processing, dynamic metabolic changes in the overall, especially for intercellular metabolism, genetic variation and environmental changes. Like the other three omics techniques, metabolomics plays an important role in systems biology. Metabolomics data sets have become more comprehensive and provide substantial evidence for the molecular mechanisms^46^.

The cellular metabolism of microbial cell responds very quickly, generating marked changes in intracellular metabolites as low as 0.1 mM/s^47–49^. The quenching of metabolism is an important precondition for accurate measurement of the concentrations of identified metabolites. The bacterial membrane and cell wall might damage during quenching causing the leakage of intracellular metabolites^50^. As a gram-negative bacterium, the cell wall structure of RA is narrower and less robust compared with yeasts and gram-positive bacteria. It seems that RA may be very susceptible to the leakages during quenching. In this study, we choose cold methanol as a quenching solution. Several studies have revealed that cold methanol is the currently most used quenching solution allowing removal of extracellular metabolites^51–54^.

After quenching and chemical derivatization, over a hundred GC–MS peaks were detected and 81 intracellular metabolites of the two organisms in the TSB medium were identified. In this study, we reveal compositional differences by multivariate analysis. The PCA model of RA is very useful to detect outliers, but the PCA score plot does not give a good representation of the class difference between the groups of two strains. For PCA, the group separations are exposed only when the inter-class variation is more than the intra-class variation. The group differentiation was influenced by many factors, like sample preparation problems^55^, experimental deviation^56^ and inadequate data pretreatments^57^. In contrast with PCA, PLS aggressively over-fit models to the sets and contributed scores in which groups are separated^58^. Meanwhile, validation is a critical step in guarantying the quality of PLS model^59^. As a result, PLS-DA analysis generates excellent group separation of two strains samples.

The 81 metabolites include organic acids, nucleotides, phosphates, amino acids, sugars, fatty acids, polyol and amine. We detected a few new metabolites that had not been shown before in *Flavobacteriaceae*, such as Inositol 1-phosphate (Ins-1P). Ins-1P is primarily formed by Myo-inositol-phosphate synthase (MIPS) catalyzing glucose-6-phosphate (Glc-6P)^60^, which is an important precursor substance of phosphatidyl-*myo*-inositol and mycothiol^61^. Ins-1P is an essential metabolite for growth and virulence in some bacteria^62,63^.

Putrescine as only one polyamine was identified in two strains. Putrescine is mainly related to biofilm formation^64^ and protein synthesis^65^, so putrescine metabolic pathways may be considered as a potential novel target for antibiotics^66–68^. The metabolism of putrescine biosynthesis in microorganisms has two alternative pathways. The first putrescine biosynthesis pathway begins with the conversion of ornithine to putrescine by ornithine decarboxylase (ODC, EC 4.1.1.17), which is encoded by *speC* gene. Another one is synthesized from Arginine by the reactions which are catalyzed by the enzymes Arginine decarboxylase (ADC, EC 4.1.1.19) and Agmatinase (EC 3.5.3.11). Ornithine is also detected in this study. It seems that the second pathway exists in RA. In Fig. 2, the variation trend of Ornithine does not correlate with Putrescine. Meanwhile, the result of ODC and ADC enzyme active is negative and positive by decarboxylase test (data not shown in this study), respectively. It suggests that the metabolite profiles provide insight and inferences into a lot of information quickly, but it remains for the analysis.

On the other hand, here, we have shown that the cellular metabolism of purine (Fig. 4c). These pathways have a pivotal role in the control of the intracellular nucleotides concentration. Although the concentration of Adenosine in RA-CH-2 was 2 times higher than that in RA-CH-1, the concentration of Adenine was regulated by the transcription and expression of Purine nucleoside phosphorylase. Linking the metabolomics data to preexisting transcriptomics data on RA can be useful in the next steps to explain the difference in the metabolism.

In this study, the application of metabolomics was first used to research the metabolic profiling and biomarkers of RA. This study revealed that RA-CH-1 and RA-CH-2 have different metabolic profiles. A large portion of different virulence and serotype phenotypes can be attributed to known virulence-associated secondary metabolites. Using the metabolome dataset, we obtained a classification model that differentiates RA-CH-1 and RA-CH-2 with good accuracy. Further, to establish a GSM model of two strains are useful frameworks to explore the versatility of RA and to understand the molecular pathogenesis. The combination of genomics and metabolomics has greatly promoted the research to identify interacting pathways. Metabolomics can also be used to bridge genotype to the phenotype of RA.

## Materials and methods

### Strains and growth conditions

RA wild strains CH-1 (BioSample ID: SAMN02603758) and CH-2 (BioSample ID: SAMN02602992) were isolated and keep in our lab. Briefly, two strains were grown in 500 ml flasks overnight at 37 °C and 150 rpm in 200 ml tryptic soy broth (TSB) and used as seed broth. The overnight cultures were added in a new 300 ml TSB medium and made the initial value of OD_600_ to 0.1. The flasks were incubated at 37 °C with shaking at 180 rpm. For untargeted metabolomics analysis, the samples were collected after 8 h of incubation. The growth curve was shown in Supplementary Fig. S1. The value of OD_600_ in RA is about 3.2. The medium was autoclaved at 121 °C for 15 min. 8 biological samples of each strain were separately processed in the same conditions and same culture time.

### Quenching and chemical derivatization of intracellular metabolites

For cell quenching, 200 ml cultures were rapidly transferred to centrifuge tubes containing 800 ml 60% methanol solution (− 40 °C) and maintained at − 20 °C in a refrigerated bath for 5 min. The mixture was centrifuged for 5 min at 7500 g and − 10 °C. The supernatant was removed rapidly and the cell suspension was washed twice with 10 ml cold 0.85% NaCl solution. After washing, the cell suspension was frozen at low temperature by the vacuum freeze dryer.

The 10 mg freeze-dried powder samples were extracted with 1 ml 50% methanol (− 20 °C) and vortexed for 30 s. To improve the precision of GC analysis, 60 μl 0.2 mg/ml nonadecylic acid–methanol solution and 60 μl of 10 mM d4-alanine-methanol solution were added to 1 ml extract as internal standard (IS). To ensure effective extraction, the tubes were treated by the freeze-thawing method with liquid nitrogen for 5 min and repeated 3 times. The extract was centrifuged for 10 min at 13,000*g* and 4 °C. The supernatant was blow-dried by vacuum concentration. The samples were resuspended in 60 μl 15 mg/ml methoxyamine pyridine solution and reacted for 120 min at 37 °C. 60 μl BSTFA reagent (containing 1% TMCS) was used for the derivatization reactions. The reaction mixture was derivatized within 90 min at 37 °C. After low-temperature centrifugation, the supernatant was used for GC–MS analysis.

### GC–MS analysis

To get the high-quality data in high throughput analysis, the quality control (QC) method was used to monitor analytical accuracy from the pooled samples and report the data quality as previously described^69^. The pooled mixtures were prepared by 20 μl of all the biological test samples.

The samples were analyzed by Agilent 7890A/5975C GC/MSD System. The complex mixture of compounds was separated on GC column HP-5MS (30 m × 250 μm × 0.25 μm, Agilent) coated with 5% phenyl/95% methylpolysiloxane. To separate the derivatives, the helium was the carrier gas set at a constant flow of 1 ml/min. 1 µl sample was injected by a split mode with a split ratio of 20:1 using the auto-sampler. The injection port temperature was set at 280 °C, the transfer line temperature set to 150 °C and the ion source temperature adjusted to 230 °C. The programs of temperature-rise were as followed: 60 °C initially for 2 min, increased to 300 °C at 10 °C/min and 300 °C was maintained for 5 min. The range of mass spectrometry was set from 35 to 750 (m/z) by a full-scan method.

### Genome-scale metabolic reconstruction of *R. anatipestifer*

The genomic data of RA-CH-1 (GenBank: CP003787.1) and RA-CH-2 (GenBank: CP004020.1) were re-annotation by RAST prokaryotic genome annotation server^70^. The draft metabolic models were generated by Model SEED (https://modelseed.org/genomes/) based on the gene re-annotation^71^. The growth conditions of RA are unknown, so RA is auto-completed in simulated media (Supplementary Table S7). The gene–protein-reaction (GPR) associations were available as an Excel file and showed the relationship among genes, their corresponding proteins and the reactions catalyzed by the proteins. The Excel files of the models were converted to SBML format to be fully compatible with COBRA Toolbox version 3.0 (https://opencobra.github.io/cobratoolbox/stable/)^72^. Because no detailed information on the biomass composition of RA has been found, the biomass composition was assembled in the Model SEED, which accounts for DNA, RNA, amino acids, nucleotides, cell wall and cofactors. DNA was calculated from the genome sequence of RA-CH-1 and RA-CH-2. DNA coefficients are calculated by first computing the GC content of the chromosome. Then the molar fractions of the deoxynucleotides are set according to the GC content. The other metabolites and their coefficients are approximations garnered from *E.coli* model *i*AF1260^73^. The metabolites (e.g., lipids, cofactors, cell wall components) are included only if the genome annotation includes evidence for functional roles associated with the biosynthesis or utilization of the metabolites. Lack of fully connected metabolic pathways may lead to contain multiple gaps due to incomplete or inconsistent annotations, the draft metabolic models were checked as a gap-filling process in TSB medium to allow biomass formation. After the gap-filling procedure, the mass balance of all reactions in the RA models was checked with elementary and charge balance by Check Model Mass Balance app in KBase (https://www.kbase.us/)^74^. To predict simulating biomass production, flux balance analysis (FBA) was used to verify the growth rate and the rate of production in the computational medium. The information of the medium in silicon used for FBA was listed in Supplementary Table S9.

### GC–MS data processing

For the metabolite identification, the Agilent GC–MS 5975 Data Analysis software was used to convert the raw GC–MS data into NetCDF format. The peaks were identified, filtered and aligned via XCMS software version 1.42.0 using XCMS's default settings with the following changes: xcmsSet (fwhm = 3, snthresh = 3, mzdiff = 0.5, step = 0.1, steps = 2, max = 300), group (bw = 2, minfrac = 0.3, max = 300)^75^. Metabolites were annotated using the Automatic Mass Spectral Deconvolution and Identification System (AMIDS) version 2.73 based on Wiley Registry and National Institute of Standards and Technology (NIST). Metabolites were confirmed via comparison of mass spectra and retention indices to the Golm Metabolome Database (GMD, http://gmd.mpimp-golm.mpg.de/) using a cut-off value of 70%. The signal integration area of each metabolite was normalized to the internal standard (Nonadecylic acid and d4-Alanine) for each sample.

### Statistical analysis

The normalized data were checked by mean-centering and unit variance (UV) scaling methods before the multivariate statistical analysis. The PCA and PLS-DA were applied to the normalized samples of the intracellular metabolite by the Simca-P version 13.0 software (Umetrics, Kinnelon). Cross-validation was used to check the quality of PCA and PLS-DA models, and 100 random permutations testing were carried out to guard against the over-fitting of PLS-DA models. The discriminating metabolites were obtained using a statistically significant threshold of variable influence on projection (VIP > 1.0). Values obtained from the PLS-DA model were validated via the Mann–Whitney–Wilcoxon test (p < 0.05). The metabolites with the values of p < 0.05 and VIP > 1.0 were chosen as discriminating metabolites between RA-CH-1 and RA-CH-2. HCA was performed using Euclidean distance methods and visualized by the pheatmap package version 1.0.8 in the R language. Metabolite correlation was assessed using the Pearson Correlation Coefficient and corresponding p values were also calculated using the Cor. test version 3.2.1 function in R. Identified metabolites were mapped onto general biochemical pathways according to the annotation in KEGG^76–78^. Pathway Activity Profiling (PAPi) algorithm was used to predict and compare the relative activity of different metabolic pathways by PAPi package version 1.14.0 in R.

## Supplementary Information

Supplementary Information 1.

Supplementary Information 2.

Supplementary Information 3.

Supplementary Information 4.

Supplementary Information 5.

Supplementary Information 6.

Supplementary Information 7.

Supplementary Information 8.

Supplementary Information 9.

Supplementary Information 10.

Supplementary Information 11.

Supplementary Information 12.

## Data Availability

The genome sequences described in this manuscript have been submitted to the National Center for Biotechnology Information (NCBI) under accession codes PRJNA172646 (whole genome and assembly data of RA-CH-1) and PRJNA183917 (whole genome and assembly data of RA-CH-2). Raw spectral data for measurement of RA-CH-1 and RA-CH-2 by GC–MS were uploaded to the Open Science Framework [Doi: https://doi.org/10.17605/OSF.IO/X6AMD]. The datasets generated during and/or analyzed in the current study are available from the corresponding author on reasonable request.

## References

[1] Zhang X (2017). Contribution of RaeB, a putative RND-type transporter to aminoglycoside and detergent resistance in *Riemerella anatipestifer*. Front. Microbiol..

[2] Liu M (2017). Use of natural transformation to establish an easy knockout method in *Riemerella anatipestifer*. Appl. Environ. Microbiol..

[3] Liu M (2018). Multiple genetic tools for editing the genome of *Riemerella anatipestifer* using a counterselectable marker. Appl. Microbiol. Biotechnol..

[4] He Y (2018). Cas1 and Cas2 from the Type II-C CRISPR-Cas system of *Riemerella anatipestifer* are required for spacer acquisition. Front Cell Infect. Microbiol..

[5] Liu M (2018). Roles of B739_1343 in iron acquisition and pathogenesis in *Riemerella anatipestifer* CH-1 and evaluation of the RA-CH-1DeltaB739_1343 mutant as an attenuated vaccine. PLoS One.

[6] Liu M (2019). Development of a markerless gene deletion strategy using rpsL as a counterselectable marker and characterization of the function of RA0C_1534 in *Riemerella anatipestifer* ATCC11845 using this strategy. PLoS One.

[7] Huang L (2019). Role of LptD in resistance to glutaraldehyde and pathogenicity in *Riemerella anatipestifer*. Front. Microbiol..

[8] Huang L (2019). DprA is essential for natural competence in *Riemerella anatipestifer* and has a conserved evolutionary mechanism. Front. Genet..

[9] Cheng A (2003). Epidemiology and new serotypes of *Riemerella anatipestifer* isolated from ducks in China and studies on their pathogenic characteristics. Chin. J. Vet. Sci..

[10] Li L (2012). Adhesion and invasion to duck embryo fibroblast cells by *Riemerella anatipestifer*. Poult. Sci..

[11] Liao H (2016). The detection of hemin-binding proteins in *Riemerella anatipestifer* CH-1. Curr. Microbiol..

[12] Liu J (2018). ATPase activity of GroEL is dependent on GroES and it is response for environmental stress in *Riemerella anatipestifer*. Microb. Pathog..

[13] Liu J (2019). Comparative genome-scale modelling of the pathogenic *Flavobacteriaceae* species *Riemerella anatipestifer* in China. Environ. Microbiol..

[14] Liu M (2019). New perspectives on Galleria mellonella larvae as a host model using *Riemerella anatipestifer* as a proof of concept. Infect. Immun..

[15] Liu M (2016). Investigation of TbfA in *Riemerella anatipestifer* using plasmid-based methods for gene over-expression and knockdown. Sci. Rep..

[16] Luo H (2015). Identification of ribosomal RNA methyltransferase gene ermF in *Riemerella anatipestifer*. Avian Pathol..

[17] Wang M (2017). Identification of the ferric iron utilization gene B739_1208 and its role in the virulence of *R. anatipestifer* CH-1. Vet. Microbiol..

[18] Wang X (2012). Development and application of specific polymerase chain reaction assay targeting the gyrB gene for rapid detection of *Riemerella anatipestifer*. Poult. Sci..

[19] Yi H (2017). Identification of a wza-like gene involved in capsule biosynthesis, pathogenicity and biofilm formation in *Riemerella anatipestifer*. Microb. Pathog..

[20] Yuan B, Cheng A, Wang M (2014). Characterization of *Riemerella anatipestifer* CH-1 gldJ gene and GldJ protein. Genet. Mol. Res..

[21] Zhong C (2013). Quantitative real-time PCR study of the expression and regulation of the tetracycline resistance gene in *Riemerella anatipestifer*. Poult. Sci..

[22] Zhu D (2019). First report of integrative conjugative elements in *Riemerella anatipestifer* isolates from ducks in China. Front Vet. Sci..

[23] Zhu D (2018). Various profiles of tet genes addition to tet(X) in *Riemerella anatipestifer* isolates from ducks in China. Front. Microbiol..

[24] Hu Q (2011). OmpA is a virulence factor of *Riemerella anatipestifer*. Vet. Microbiol..

[25] Huang L (2017). Type B chloramphenicol acetyltransferases are responsible for chloramphenicol resistance in *Riemerella anatipestifer*, China. Front. Microbiol..

[26] Liao H (2015). TonB energy transduction systems of *Riemerella anatipestifer* are required for iron and hemin utilization. PLoS One.

[27] Luo H (2018). A novel resistance gene, lnu(H), conferring resistance to lincosamides in *Riemerella anatipestifer* CH-2. Int. J. Antimicrob. Agents..

[28] Wang X (2016). The *Riemerella anatipestifer* AS87_01735 gene encodes nicotinamidase PncA, an important virulence factor. Appl. Environ. Microbiol..

[29] Passalacqua KD, Charbonneau ME, O'Riordan M (2016). Bacterial metabolism shapes the host-pathogen interface. Microbiol. Spectr..

[30] Hinz KH, Ryll M, Kohler B (1998). Detection of acid production from carbohydrates by *Riemerella anatipestifer* and related organisms using the buffered single substrate test. Vet. Microbiol..

[31] Piechulla K, Pohl S, Mannheim W (1986). Phenotypic and genetic relationships of so-called *Moraxella* (*Pasteurella*) *anatipestifer* to the Flavobacterium/Cytophaga group. Vet. Microbiol..

[32] Segers P (1993). *Riemerella anatipestifer* gen. nov., comb. Nov., the causative agent of septicemia anserum exudativa, and its phylogenic affiliation within the Flavobacterium-Cytophaga rRNA homology group. Int. J. Syst. Bacteriol..

[33] Tang Y, Ji-Xiang LI, Gao JY, Ding MJ, Zhu ZF (2010). Purification and characterization of gelatinase from *Riemerella anatipestifer* strain AF, China. Anim. Husband. Vet. Med..

[34] Planchon M, Leger T, Spalla O, Huber G, Ferrari R (2017). Metabolomic and proteomic investigations of impacts of titanium dioxide nanoparticles on *Escherichia coli*. PLoS One.

[35] Liu J (2016). Genome-wide analysis of the synonymous codon usage patterns in *Riemerella anatipestifer*. Int. J. Mol. Sci..

[36] Liu M (2017). Identifying the genes responsible for iron-limited condition in *Riemerella anatipestifer* CH-1 through RNA-seq-based analysis. Biomed. Res. Int..

[37] Wang X (2014). Comparative genomics of *Riemerella anatipestifer* reveals genetic diversity. BMC Genom..

[38] Wang X (2012). Complete genome sequence of *Riemerella anatipestifer* reference strain. J. Bacteriol..

[39] Zhu D (2016). Comparative genomic analysis identifies structural features of CRISPR-Cas systems in *Riemerella anatipestifer*. BMC Genom..

[40] Gao Q (2016). Development of an indirect ELISA using recombinant ompH protein for serological detection of *Riemerella anatipestifer* infection in ducks. Int. J. Clin. Exp. Med..

[41] Zhong C (2009). Antibiotic susceptibility of *Riemerella anatipestifer* field isolates. Avian Dis..

[42] Patti GJ, Yanes O, Siuzdak G (2012). Innovation: Metabolomics: The apogee of the omics trilogy. Nat. Rev. Mol. Cell Biol..

[43] Schrimpe-Rutledge AC, Codreanu SG, Sherrod SD, McLean JA (2016). Untargeted metabolomics strategies-challenges and emerging directions. J. Am. Soc. Mass. Spectrom..

[44] Papadimitropoulos MP, Vasilopoulou CG, Maga-Nteve C, Klapa MI (2018). Untargeted GC-MS metabolomics. Methods Mol. Biol..

[45] Aggio RB, Ruggiero K, Villas-Boas SG (2010). Pathway activity profiling (PAPi): From the metabolite profile to the metabolic pathway activity. Bioinformatics.

[46] Aurich MK (2015). Prediction of intracellular metabolic states from extracellular metabolomic data. Metabolomics.

[47] Buckstein MH, He J, Rubin H (2008). Characterization of nucleotide pools as a function of physiological state in *Escherichia coli*. J. Bacteriol..

[48] Usuda Y (2010). Dynamic modeling of *Escherichia coli* metabolic and regulatory systems for amino-acid production. J. Biotechnol..

[49] Yukihira D, Miura D, Saito K, Takahashi K, Wariishi H (2010). MALDI-MS-based high-throughput metabolite analysis for intracellular metabolic dynamics. Anal. Chem..

[50] Bolten CJ, Kiefer P, Letisse F, Portais JC, Wittmann C (2007). Sampling for metabolome analysis of microorganisms. Anal. Chem..

[51] Faijes M, Mars AE, Smid EJ (2007). Comparison of quenching and extraction methodologies for metabolome analysis of *Lactobacillus plantarum*. Microb. Cell Fact..

[52] Tian S, Wang C, Yang L, Zhang Y, Tang T (2019). Comparison of five extraction methods for intracellular metabolites of *Salmonella typhimurium*. Curr. Microbiol..

[53] Villas-Boas SG, Bruheim P (2007). Cold glycerol-saline: The promising quenching solution for accurate intracellular metabolite analysis of microbial cells. Anal. Biochem..

[54] Zhang Q (2018). Comprehensive optimization of the metabolomic methodology for metabolite profiling of *Corynebacterium glutamicum*. Appl. Microbiol. Biotechnol..

[55] Vuckovic D (2012). Current trends and challenges in sample preparation for global metabolomics using liquid chromatography-mass spectrometry. Anal. Bioanal. Chem..

[56] Teahan O (2006). Impact of analytical bias in metabonomic studies of human blood serum and plasma. Anal. Chem..

[57] van den Berg RA, Hoefsloot HC, Westerhuis JA, Smilde AK, van der Werf MJ (2006). Centering, scaling, and transformations: Improving the biological information content of metabolomics data. BMC Genom..

[58] Szymanska E, Saccenti E, Smilde AK, Westerhuis JA (2012). Double-check: Validation of diagnostic statistics for PLS-DA models in metabolomics studies. Metabolomics.

[59] Worley B, Powers R (2013). Multivariate analysis in metabolomics. Curr. Metab..

[60] Huang X, Hernick M (2015). Recombinant expression of a functional myo-inositol-1-phosphate synthase (MIPS) in *Mycobacterium smegmatis*. Protein J..

[61] Morita YS (2011). Inositol lipid metabolism in mycobacteria: Biosynthesis and regulatory mechanisms. Biochim. Biophys. Acta.

[62] Chen C (2019). Myo-inositol-1-phosphate synthase (Ino-1) functions as a protection mechanism in *Corynebacterium glutamicum* under oxidative stress. Microbiologyopen.

[63] Movahedzadeh F (2004). The *Mycobacterium tuberculosis* ino1 gene is essential for growth and virulence. Mol. Microbiol..

[64] Patel CN (2006). Polyamines are essential for the formation of plague biofilm. J. Bacteriol..

[65] Igarashi K, Kashiwagi K (2000). Polyamines: Mysterious modulators of cellular functions. Biochem. Biophys. Res. Commun..

[66] Guerra PR (2018). Putrescine biosynthesis and export genes are essential for normal growth of avian pathogenic *Escherichia coli*. BMC Microbiol..

[67] Jelsbak L (2014). Identification of metabolic pathways essential for fitness of *Salmonella Typhimurium* in *vivo*. PLoS One.

[68] Shah P, Nanduri B, Swiatlo E, Ma Y, Pendarvis K (2011). Polyamine biosynthesis and transport mechanisms are crucial for fitness and pathogenesis of *Streptococcus pneumoniae*. Microbiology.

[69] Sangster T, Major H, Plumb R, Wilson AJ, Wilson ID (2006). A pragmatic and readily implemented quality control strategy for HPLC-MS and GC-MS-based metabonomic analysis. Analyst.

[70] Overbeek R (2014). The SEED and the rapid annotation of microbial genomes using subsystems technology (RAST). Nucleic Acids Res..

[71] Henry CS (2010). High-throughput generation, optimization and analysis of genome-scale metabolic models. Nat. Biotechnol..

[72] Heirendt L (2019). Creation and analysis of biochemical constraint-based models using the COBRA Toolbox v.3.0. Nat. Protoc..

[73] Feist AM (2007). A genome-scale metabolic reconstruction for *Escherichia coli* K-12 MG1655 that accounts for 1260 ORFs and thermodynamic information. Mol. Syst. Biol..

[74] Arkin AP (2018). KBase: The United States Department of Energy Systems Biology Knowledgebase. Nat. Biotechnol..

[75] Smith CA, Want EJ, O'Maille G, Abagyan R, Siuzdak G (2006). XCMS: Processing mass spectrometry data for metabolite profiling using nonlinear peak alignment, matching, and identification. Anal. Chem..

[76] Kanehisa M, Goto S (2000). KEGG: Kyoto encyclopedia of genes and genomes. Nucleic Acids Res..

[77] Kanehisa M, Sato Y, Furumichi M, Morishima K, Tanabe M (2019). New approach for understanding genome variations in KEGG. Nucleic Acids Res..

[78] Kanehisa M (2019). Toward understanding the origin and evolution of cellular organisms. Protein Sci..

