# Supplementary figures and images for: Comparative genomics and metabolomics analysis of Riemerella anatipestifer strain CH-1 and CH-2
Source: Sci Rep. 2021 Jan 12;11:616. doi: 10.1038/s41598-020-79733-w (PMC7804117; doi:10.1038/s41598-020-79733-w)

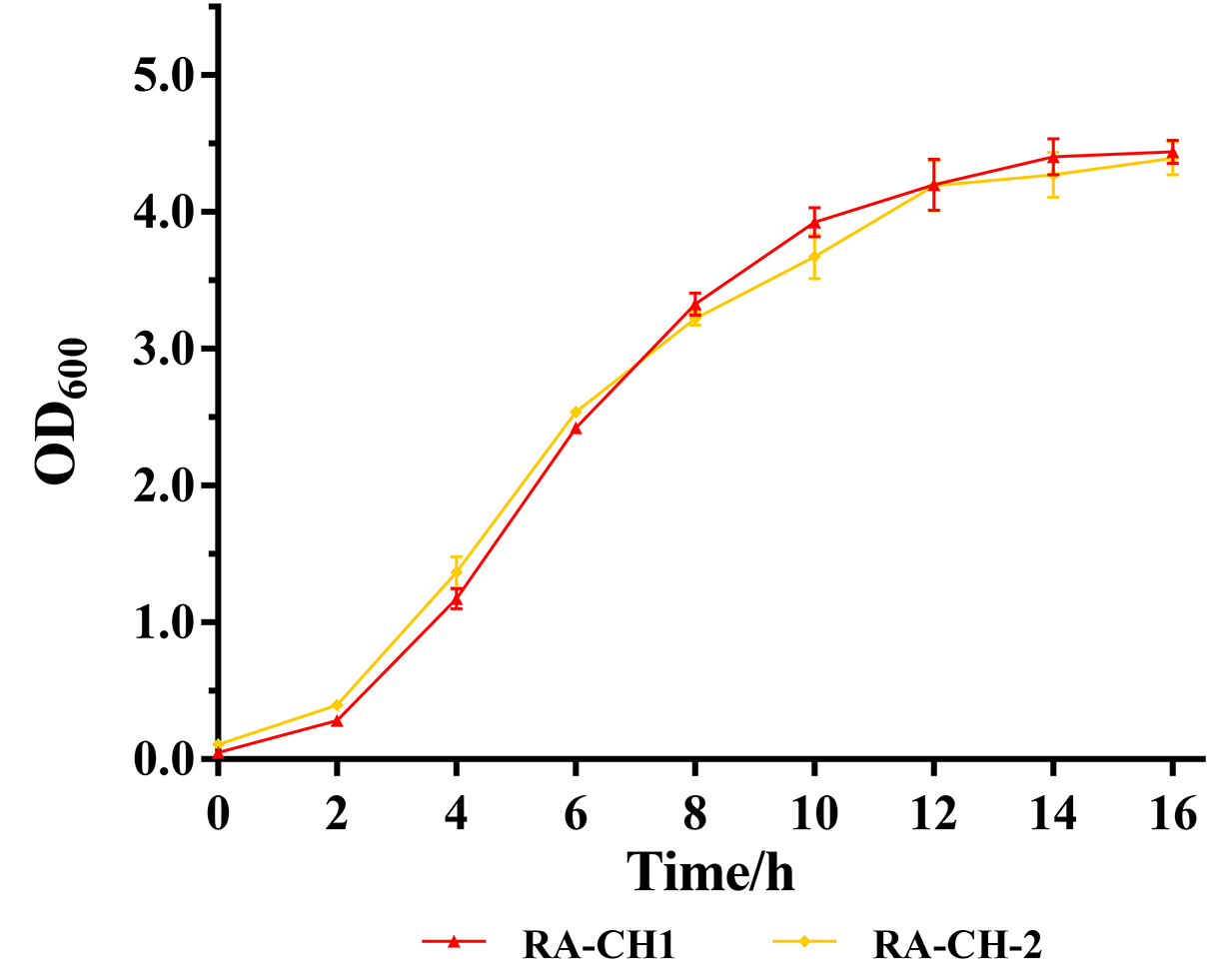

Supplement: Supplementary file 1 — Supplementary Information 1. [file 41598_2020_79733_MOESM1_ESM.tif]

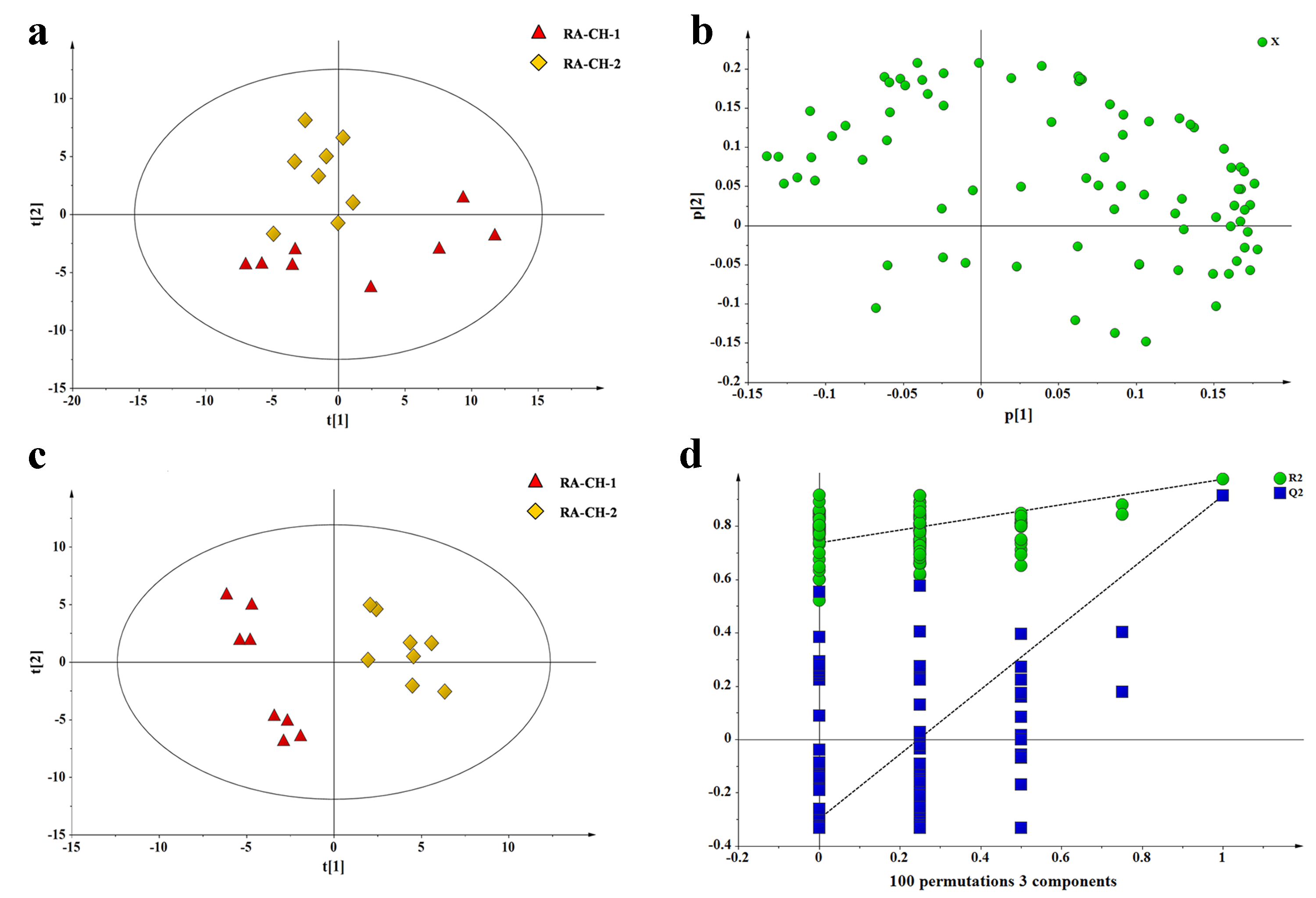

Supplement: Supplementary file 2 — Supplementary Information 2. [file 41598_2020_79733_MOESM2_ESM.tif]
