# Supplementary material for: Comparative genomics and metabolomics analysis of Riemerella anatipestifer strain CH-1 and CH-2
Source: Sci Rep. 2021 Jan 12;11:616. doi: 10.1038/s41598-020-79733-w (PMC7804117; doi:10.1038/s41598-020-79733-w)
Supplement: Supplementary file 3 — Supplementary Information 3. [file 41598_2020_79733_MOESM3_ESM.docx]

**Table S1.** The biomass composition of RA-CH-1 and RA-CH-2 cells

| **Compound ID** | **Compound Name** | **Coefficient** | **Compartment*** |
| --- | --- | --- | --- |
| cpd00001 | H_2_O | -35.54030924 | c0 |
| cpd00002 | ATP | -40.11017574 | c0 |
| cpd00003 | NAD | -0.003096467 | c0 |
| cpd00006 | NADP | -0.003096467 | c0 |
| cpd00008 | ADP | 40 | c0 |
| cpd00009 | Phosphate | 39.99690353 | c0 |
| cpd00010 | CoA | -0.003096467 | c0 |
| cpd00012 | PPi | 0.484610636 | c0 |
| cpd00015 | FAD | -0.003096467 | c0 |
| cpd00016 | Pyridoxal phosphate | -0.003096467 | c0 |
| cpd00017 | S-Adenosyl-L-methionine | -0.003096467 | c0 |
| cpd00023 | L-Glutamate | -0.219088153 | c0 |
| cpd00028 | Heme | -0.003096467 | c0 |
| cpd00030 | Mn^2+^ | -0.003096467 | c0 |
| cpd00033 | Glycine | -0.509869787 | c0 |
| cpd00034 | Zn^2+^ | -0.003096467 | c0 |
| cpd00035 | L-Alanine | -0.42793438 | c0 |
| cpd00038 | GTP | -0.135406821 | c0 |
| cpd00039 | L-Lysine | -0.28543802 | c0 |
| cpd00041 | L-Aspartate | -0.200830807 | c0 |
| cpd00042 | GSH | -0.003096467 | c0 |
| cpd00048 | Sulfate | -0.003096467 | c0 |
| cpd00051 | L-Arginine | -0.246696823 | c0 |
| cpd00052 | CTP | -0.084103616 | c0 |
| cpd00053 | L-Glutamine | -0.219088153 | c0 |
| cpd00054 | L-Serine | -0.179456353 | c0 |
| cpd00056 | TPP | -0.003096467 | c0 |
| cpd00058 | Cu^2+^ | -0.003096467 | c0 |
| cpd00060 | L-Methionine | -0.127801423 | c0 |
| cpd00062 | UTP | -0.090831905 | c0 |
| cpd00063 | Ca^2+^ | -0.003096467 | c0 |
| cpd00065 | L-Tryptophan | -0.047201919 | c0 |
| cpd00066 | L-Phenylalanine | -0.15451949 | c0 |
| cpd00067 | H^+^ | 40 | c0 |
| cpd00069 | L-Tyrosine | -0.120676605 | c0 |
| cpd00084 | L-Cysteine | -0.076146492 | c0 |
| cpd00087 | Tetrahydrofolate | -0.003096467 | c0 |
| cpd00099 | Cl^-^ | -0.003096467 | c0 |
| cpd00107 | L-Leucine | -0.375388848 | c0 |
| cpd00115 | dATP | -0.020798035 | c0 |
| cpd00118 | Putrescine | -0.003096467 | c0 |
| cpd00119 | L-Histidine | -0.0792636 | c0 |
| cpd00129 | L-Proline | -0.184354665 | c0 |
| cpd00132 | L-Asparagine | -0.200830807 | c0 |
| cpd00149 | Co^2+^ | -0.003096467 | c0 |
| cpd00156 | L-Valine | -0.352233189 | c0 |
| cpd00161 | L-Threonine | -0.211072733 | c0 |
| cpd00166 | Calomide | -0.003096467 | c0 |
| cpd00201 | 10-Formyltetrahydrofolate | -0.003096467 | c0 |
| cpd00205 | K^+^ | -0.003096467 | c0 |
| cpd00220 | Riboflavin | -0.003096467 | c0 |
| cpd00241 | dGTP | -0.011248244 | c0 |
| cpd00254 | Mg^2+^ | -0.003096467 | c0 |
| cpd00264 | Spermidine | -0.003096467 | c0 |
| cpd00322 | L-Isoleucine | -0.24179851 | c0 |
| cpd00345 | 5-Methyltetrahydrofolate | -0.003096467 | c0 |
| cpd00356 | dCTP | -0.011248244 | c0 |
| cpd00357 | TTP | -0.020798035 | c0 |
| cpd00557 | Siroheme | -0.003096467 | c0 |
| cpd01997 | Dimethylbenzimidazole | 0.003096467 | c0 |
| cpd02229 | Bactoprenyl diphosphate | -0.025010598 | c0 |
| cpd03422 | Cobinamide | 0.003096467 | c0 |
| cpd10515 | Fe^2+^ | -0.003096467 | c0 |
| cpd10516 | Fe^3+^ | -0.003096467 | c0 |
| cpd11416 | Biomass | 1 | c0 |
| cpd11493 | ACP | -0.003096467 | c0 |
| cpd12370 | apo-ACP | 0.003096467 | c0 |
| cpd15352 | 2-Demethylmenaquinone 8 | -0.003096467 | c0 |
| cpd15432 | core oligosaccharide lipid A | -0.025010598 | c0 |
| cpd15500 | Menaquinone 8 | -0.003096467 | c0 |
| cpd15533 | phosphatidylethanolamine dioctadecanoyl | -0.010648042 | c0 |
| cpd15540 | Phosphatidylglycerol dioctadecanoyl | -0.010648042 | c0 |
| cpd15560 | Ubiquinone-8 | -0.003096467 | c0 |
| cpd15665 | Peptidoglycan polymer (n subunits) | -0.025010598 | c0 |
| cpd15666 | Peptidoglycan polymer (n-1 subunits) | 0.025010598 | c0 |
| cpd15695 | Diisoheptadecanoylphosphatidylethanolamine | -0.010648042 | c0 |
| cpd15696 | Dianteisoheptadecanoylphosphatidylethanolamine | -0.010648042 | c0 |
| cpd15722 | Diisoheptadecanoylphosphatidylglycerol | -0.010648042 | c0 |
| cpd15723 | Dianteisoheptadecanoylphosphatidylglycerol | -0.010648042 | c0 |
| cpd15793 | Stearoylcardiolipin | -0.010648042 | c0 |
| cpd15794 | Isoheptadecanoylcardiolipin | -0.010648042 | c0 |
| cpd15795 | Anteisoheptadecanoylcardiolipin | -0.010648042 | c0 |
| cpd17041 | Protein biosynthesis | -1 | c0 |
| cpd17042 | DNA replication | -1 | c0 |
| cpd17043 | RNA transcription | -1 | c0 |

* c0: Cytosol
