# Supplementary material for: Comparative genomics and metabolomics analysis of Riemerella anatipestifer strain CH-1 and CH-2
Source: Sci Rep. 2021 Jan 12;11:616. doi: 10.1038/s41598-020-79733-w (PMC7804117; doi:10.1038/s41598-020-79733-w)
Supplement: Supplementary file 9 — Supplementary Information 9. [file 41598_2020_79733_MOESM9_ESM.docx]

**Table S7.** Principal components factor loadings for 81 metabolites from RA.

| Metabolite | p[1] ^a^ | p[2]^b^ |
| --- | --- | --- |
| Pyruvic acid | 0.102059 | -0.049795 |
| Lactic acid | 0.0625328 | -0.0267532 |
| Glycolic acid | -0.0244071 | -0.0409384 |
| Alanine | 0.151461 | 0.0106712 |
| Heptanoic acid | -0.0763624 | 0.0840468 |
| Monomethylphosphate | 0.173301 | 0.0261256 |
| Valine | 0.156653 | 0.0981028 |
| Urea | -0.0674538 | -0.105597 |
| Leucine | 0.137264 | 0.124885 |
| phosphoric acid | 0.16146 | 0.0735154 |
| isoleucine | 0.135199 | 0.129369 |
| Proline | 0.178172 | -0.0307975 |
| Maleic acid | 0.0232626 | -0.0526091 |
| Glycine | 0.108395 | 0.132999 |
| Succinic acid | 0.173519 | -0.0571263 |
| Glyceric acid | 0.0833215 | 0.154559 |
| Uracil | -0.0517589 | 0.187622 |
| Fumaric acid | 0.130664 | -0.00468794 |
| Nonanoic acid | -0.0621744 | 0.189748 |
| Serine | 0.039182 | 0.203706 |
| Threonine | 0.12806 | 0.137003 |
| Thymine | -0.048567 | 0.178852 |
| Homoserine | 0.0861365 | 0.0210244 |
| Putrescine | -0.0605716 | 0.108772 |
| Erythrose | 0.0757761 | 0.0508977 |
| Nicotinamide | 0.125234 | 0.0151952 |
| Malic acid | -0.0240219 | 0.194515 |
| Parabanic acid | 0.101984 | -0.0495749 |
| Methionine | 0.167219 | 0.0748675 |
| Aspartic acid | -0.0010277 | 0.207671 |
| Pyroglutamic acid | 0.169722 | 0.0690131 |
| Cytosine | 0.0456931 | 0.132078 |
| N-Acetylglutamic acid | 0.0918197 | 0.141356 |
| 2, 4, 6-Tritert-butylbenzenethiol | -0.0872743 | 0.127676 |
| Cysteine | 0.159774 | -0.062074 |
| Citramalic acid | 0.0863556 | -0.137372 |
| α-ketoglutaric acid | -0.0238958 | 0.153312 |
| Pipecolic acid | 0.0259394 | 0.0498946 |
| Glutamic acid | 0.167019 | 0.00505623 |
| Phenylalanine | 0.176381 | 0.0537876 |
| Suberyl glycine | 0.164887 | -0.045094 |
| Dodecanoic acid | 0.067954 | 0.0608636 |
| Metabolite | p[1] | p[2] |
| Asparagine | 0.127018 | -0.0571369 |
| Arabinose | 0.0795361 | 0.0867086 |
| Glycerol-2-phosphate | -0.109005 | 0.0873047 |
| Glycerol-3-phosphate | 0.129621 | 0.033842 |
| Glutamine | 0.149395 | -0.06199 |
| Tetradecanoic acid | -0.109934 | 0.145954 |
| Hypoxanthine | 0.161093 | -0.00104909 |
| Glyceric acid-3-phosphate | 0.0916124 | 0.115413 |
| Ornithine | 0.0197753 | 0.188283 |
| Citric acid | 0.171927 | -0.00780464 |
| Lysine | 0.0903021 | 0.0504156 |
| Adenine | 0.0633309 | 0.183913 |
| Pentadecanoic acid | -0.106847 | 0.0572428 |
| Fructose | 0.0651395 | 0.186549 |
| Galactose | 0.0629597 | 0.19051 |
| Glucose | 0.063947 | 0.187446 |
| Tyrosine | 0.167595 | 0.0462322 |
| Sorbitol | -0.0602668 | -0.0505568 |
| Glucopyranose | -0.0340148 | 0.168218 |
| Hexadecanoic acid | -0.130476 | 0.0877192 |
| Heptadecanoic acid | -0.02501 | 0.0212275 |
| Guanine | 0.0609085 | -0.120909 |
| Tryptophan | 0.105339 | 0.0395608 |
| Octadecanoic acid | -0.137678 | 0.0887411 |
| Eicosanol | -0.0050193 | 0.0445522 |
| Fructose-6-phosphate | 0.151422 | -0.103121 |
| Glucose-6-phosphate | 0.106396 | -0.147948 |
| myo-Inositol-1-phosphate | 0.163206 | 0.0253341 |
| Uridine | -0.0407628 | 0.207359 |
| 2-Monopalmitoylglycerol | -0.0583708 | 0.144666 |
| 1-Monohexadecanoylglycerol | -0.118328 | 0.0610434 |
| Adenosine | -0.0375202 | 0.185669 |
| Sucrose | -0.0098798 | -0.0478991 |
| 2-Monostearoylglycerol | -0.0958594 | 0.114007 |
| 1-Monooctadecanoylglycerol | -0.126988 | 0.0536821 |
| Trehalose | 0.16976 | 0.0201751 |
| Guanosine | -0.0588084 | 0.18255 |
| Isomaltose | 0.165915 | 0.046326 |
| Adenosine-5-monophosphate | 0.170138 | -0.0284438 |

^a^ p[1]: the contributor of the first component.

^b^ p[2]: the contributor of the second component.
