# Supplementary material for: Comparative genomics and metabolomics analysis of Riemerella anatipestifer strain CH-1 and CH-2
Source: Sci Rep. 2021 Jan 12;11:616. doi: 10.1038/s41598-020-79733-w (PMC7804117; doi:10.1038/s41598-020-79733-w)
Supplement: Supplementary file 11 — Supplementary Information 11. [file 41598_2020_79733_MOESM11_ESM.docx]

**Table S9.** The in silico (computational) media

| **Name** | **ID** | **Concentration** | **Min Flux** | **Max Flux** |
| --- | --- | --- | --- | --- |
| H_2_O | cpd00001 | 0.001 | -100 | 100 |
| O_2_ | cpd00007 | 0.001 | -100 | 100 |
| Phosphate | cpd00009 | 0.001 | -100 | 100 |
| AMP | cpd00018 | 0.001 | -100 | 100 |
| L-Glutamate | cpd00023 | 0.001 | -100 | 100 |
| D-Glucose | cpd00027 | 0.001 | -100 | 100 |
| Heme | cpd00028 | 0.001 | -100 | 100 |
| Mn_2+_ | cpd00030 | 0.001 | -100 | 100 |
| Glycine | cpd00033 | 0.001 | -100 | 100 |
| Zn^2+^ | cpd00034 | 0.001 | -100 | 100 |
| L-Alanine | cpd00035 | 0.001 | -100 | 100 |
| L-Lysine | cpd00039 | 0.001 | -100 | 100 |
| L-Aspartate | cpd00041 | 0.001 | -100 | 100 |
| CMP | cpd00046 | 0.001 | -100 | 100 |
| Sulfate | cpd00048 | 0.001 | -100 | 100 |
| L-Arginine | cpd00051 | 0.001 | -100 | 100 |
| L-Serine | cpd00054 | 0.001 | -100 | 100 |
| Cu^2+^ | cpd00058 | 0.001 | -100 | 100 |
| L-Methionine | cpd00060 | 0.001 | -100 | 100 |
| Ca^2+^ | cpd00063 | 0.001 | -100 | 100 |
| L-Tryptophan | cpd00065 | 0.001 | -100 | 100 |
| L-Phenylalanine | cpd00066 | 0.001 | -100 | 100 |
| H^+^ | cpd00067 | 0.001 | -100 | 100 |
| L-Tyrosine | cpd00069 | 0.001 | -100 | 100 |
| L-Cysteine | cpd00084 | 0.001 | -100 | 100 |
| UMP | cpd00091 | 0.001 | -100 | 100 |
| Uracil | cpd00092 | 0.001 | -100 | 100 |
| Cl^-^ | cpd00099 | 0.001 | -100 | 100 |
| L-Leucine | cpd00107 | 0.001 | -100 | 100 |
| L-Histidine | cpd00119 | 0.001 | -100 | 100 |
| GMP | cpd00126 | 0.001 | -100 | 100 |
| L-Proline | cpd00129 | 0.001 | -100 | 100 |
| Co^2+^ | cpd00149 | 0.001 | -100 | 100 |
| L-Valine | cpd00156 | 0.001 | -100 | 100 |
| L-Threonine | cpd00161 | 0.001 | -100 | 100 |
| Adenosine | cpd00182 | 0.001 | -100 | 100 |
| Thymidine | cpd00184 | 0.001 | -100 | 100 |
| K^+^ | cpd00205 | 0.001 | -100 | 100 |
| Pyridoxal | cpd00215 | 0.001 | -100 | 100 |
| Niacin | cpd00218 | 0.001 | -100 | 100 |
| Prephenate | cpd00219 | 0.001 | -100 | 100 |
| Riboflavin | cpd00220 | 0.001 | -100 | 100 |
| HYXN | cpd00226 | 0.001 | -100 | 100 |
| H_2_S | cpd00239 | 0.001 | -100 | 100 |
| Inosine | cpd00246 | 0.001 | -100 | 100 |
| Uridine | cpd00249 | 0.001 | -100 | 100 |
| Mg^2+^ | cpd00254 | 0.001 | -100 | 100 |
| Guanosine | cpd00311 | 0.001 | -100 | 100 |
| L-Isoleucine | cpd00322 | 0.001 | -100 | 100 |
| L-Cystine | cpd00381 | 0.001 | -100 | 100 |
| Shikimate | cpd00383 | 0.001 | -100 | 100 |
| Folate | cpd00393 | 0.001 | -100 | 100 |
| Deoxyadenosine | cpd00438 | 0.001 | -100 | 100 |
| Lipoate | cpd00541 | 0.001 | -100 | 100 |
| PAN | cpd00644 | 0.001 | -100 | 100 |
| Deoxycytidine | cpd00654 | 0.001 | -100 | 100 |
| Thiamine phosphate | cpd00793 | 0.001 | -100 | 100 |
| Na^+^ | cpd00971 | 0.001 | -100 | 100 |
| Arsenate | cpd01048 | 0.001 | -100 | 100 |
| Vitamin B12 | cpd03424 | 0.001 | -100 | 100 |
| Fe^2+^ | cpd10515 | 0.001 | -100 | 100 |
| Fe^3+^ | cpd10516 | 0.001 | -100 | 100 |
| Chromate | cpd11595 | 0.001 | -100 | 100 |
